# Sticky Science: Using Complex Coacervate Adhesives for Biomedical Applications

**DOI:** 10.1002/adhm.202402340

**Published:** 2024-10-01

**Authors:** Ayla N. Kwant, Julien S. Es Sayed, Marleen Kamperman, Janette K. Burgess, Dirk‐Jan Slebos, Simon D. Pouwels

**Affiliations:** ^1^ Department of Pulmonary Diseases University Medical Center Groningen Hanzeplein 1 Groningen 9713GZ The Netherlands; ^2^ Polymer Science Zernike Institute for Advanced Materials (ZIAM) University of Groningen Nijenborgh 3 Groningen 9747AG The Netherlands; ^3^ Department of Pathology and Medical Biology University Medical Center Groningen Hanzeplein 1 Groningen 9713GZ The Netherlands; ^4^ Groningen Research Institute for Asthma and COPD University Medical Center Groningen Hanzeplein 1 Groningen 9713GZ The Netherlands

**Keywords:** complex coacervate, embolic agent, glue, hemostat, medical adhesive, polyelectrolyte, sealant

## Abstract

Tissue adhesives are used for various medical applications, including wound closure, bleeding control, and bone healing. Currently available options often show weak adhesion or cause adverse effects. Recently, there has been an increasing interest in complex coacervates as medical adhesives. Complex coacervates are formed by mixing oppositely charged macromolecules that associate and undergo liquid‐liquid phase separation, in which the dense bottom phase is the complex coacervate. Complex coacervates are strong and often biocompatible, and show strong underwater adhesion. The properties of the resulting materials are tunable by intrinsic factors such as polymer chemistry, molecular weight, charge density, and topology of the macromolecules, as well as extrinsic factors such as temperature, pH, and salt concentration. Therefore, complex coacervates are interesting new candidates for medical adhesives. In this review, it is described how complex coacervates form and how different factors influence their behavior. Next, an overview of recent studies on complex coacervates in the context of medical adhesives is presented. The application of complex coacervates as hemostatic or embolic agents, skin or bone repair adhesives, and soft tissue sealants is discussed. Lastly, additional possibilities for utilizing these materials in the future are discussed.

## Graphical abstract

Complex coacervates have shown to be promising candidates to replace currently available medical adhesives. Their biocompatibility, strong underwater adhesion, tunable, and stimuli‐responsive properties make them suitable for application as hemostatic and embolic agents, skin and bone repair adhesives, and as soft tissue sealant. These materials show potential for many future applications as medical adhesives.

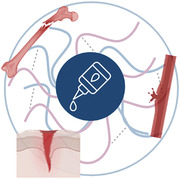

## 1 Introduction

Tissue adhesives are versatile materials that can be used for various medical applications, like wound closure, bleeding control after surgical procedures. An adhesive can be an alternative to invasive medical tools like sutures, staples, and wires. Currently, medical adhesives mostly consist of cyanoacrylates, which are fast‐acting adhesives that polymerize upon contact with moisture.^[^ ^1^ ^]^ While they are strong adhesives, they are often associated with cytotoxicity and are incompatible with usage on wet surfaces.^[^ ^2^ ^]^ Furthermore, cyanoacrylates are stiff and brittle, which does not match the mechanical properties of the mostly soft and dynamic tissues of the human body.^[^ ^3^ ^]^ Another important group of tissue adhesives are natural adhesives, such as fibrin‐ and albumin‐based sealants. While these are less cytotoxic, they are associated with poor bonding strength, fast degradation, and allergic reactions.^[^ ^4^ ^]^ An overview of the most commonly used medical adhesives, their mechanism of action and adverse effects can be found in **Table** **1**. Due to the many shortcomings, there is a strong need for the development of novel medical tissue adhesives that are bio‐compatible but also have favorable mechanical properties, and can be used in a soft, dynamic, and wet environment. Strong efforts have been made in order to develop new types of strong wet adhesives, for example by using hydrogels.^[^ ^5^ ^]^

**Table 1:** Commonly used medical adhesives and sealants.

	Applications	Mechanism of action	Examples	Adverse effects and shortcomings
*Natural*				
Fibrin^[^ ^14^, ^15^, ^16^ ^]^	Hemostat, wound healing, tissue sealant	Fibrinogen and thrombin are administered, fibrinogen is converted to fibrin. Fibrin forms a clot with adhesive properties.	Artiss (Baxter), Tisseel (Baxter), Evicel (Ethicon, J&J)	Hypotension, blood‐borne disease, anaphylaxis
Albumin^[^ ^15^, ^16^, ^17^ ^]^	Tissue sealant	Albumin is cross‐linked to ECM and cell surfaces using glutaraldehyde or polyaldehyde.	BioGlue (Cryolife), Preveleak (Baxter)	Anaphylaxis, inflammation, necrosis, edema
Collagen^[^ ^15^, ^16^, ^18^ ^]^	Wound closure, tissue sealant, hemostat	Collagen activates the clot formation cascade, initiating wound healing.	TachoSil (Baxter), Vitagel (Orthovita/Stryker), FloSeal (Baxter)	Infection, immune response, swelling
*Synthetic*				
Cyanoacrylate^[^ ^1^, ^15^, ^16^ ^]^	Wound healing, tissue sealant, embolic agent	Liquid monomers that polymerize upon exposure to moisture.	Dermabond (Ethicon, J&J), Histoacryl (B. Braun Medical Inc.)	Inflammation, impaired wound healing, thrombosis
Polyurethane^[^ ^15^, ^16^, ^19^ ^]^	Wound healing	Prepolymers react with moisture, forming covalent bonds with the material's surface.	TissuGlu	Hematoma, wound necrosis, long setting time
Polyethylene glycol^[^ ^15^, ^20^ ^]^	Wound healing, tissue sealant, bone repair	PEG forms an adhesive hydrogel upon photoactivation or addition of another solution.	CoSeal (Baxter), FocalSeal (Focal Inc.), DuraSeal (Covidien Inc.)	Swelling of the gel, low cohesive strength, toxicity from UV or cross‐linker

Recently, a new class of adhesives called complex coacervates was discovered, inspired by the strong underwater adhesion of the sandcastle worm (*Phragmatopoma californica*).^[^ ^6^ ^]^ These adhesives are soft and water‐based, making them good candidates for medical adhesives. Recent studies have investigated the use of complex coacervate adhesives to treat a wide range of defects and diseases, including skin wounds, hemorrhage, and bone fractures.^[^ ^7^, ^8^, ^9^ ^]^ The mechanical and adhesive properties of complex coacervates, in relation to their composition and structure have previously been reviewed.^[^ ^10^, ^11^ ^]^ The more complex dynamics of these systems have also been described elsewhere.^[^ ^12^, ^13^ ^]^ However, an overview of the diverse medical applications of these materials is lacking. In this review, the concept of complex coacervates, how they are formed, and how their properties can be controlled will be discussed, as well as how they can be utilized. Several medical applications will be described, focusing on the most recent developments. The complex coacervate medical adhesives will be divided into five classes based on their application: bleeding control (hemostats), skin repair, bone repair, sealants, and blocking blood flow (embolic agents). Lastly, a future perspective will be given of possible additional applications and improvements, as well as suggestions to overcome the current limitations of these adhesives.

## 2 Complex Coacervate Formation and Properties

**Figure 1:**
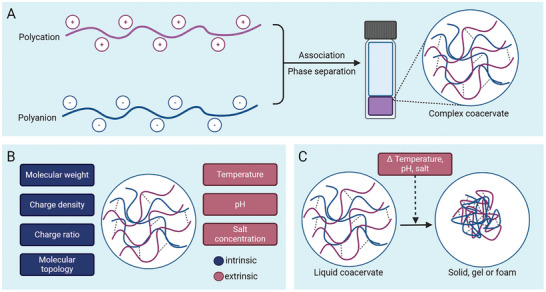
a) Illustration of the formation of complex coacervates. b) Intrinsic and extrinsic factors that influence the material properties of complex coacervates. c) Liquid‐to‐solid transition of complex coacervates as a result of changes in temperature, pH, or salt concentration. Figure was created with BioRender.com.

Complex coacervates are formed through liquid‐liquid phase separation (LLPS) that results from the association of two species of macromolecules (large molecules such as polymers, proteins, polysaccharides) in an aqueous solution (**Figure** **1**A).^[^ ^11^, ^21^ ^]^ This review focuses on complex coacervates specifically, as opposed to simple coacervates, which are formed through LLPS driven by only one species of macromolecules.^[^ ^22^ ^]^ The complex coacervation process produces a two‐phase system composed of a dense macromolecule‐rich phase, which is the complex coacervate, in equilibrium with a polymer‐depleted phase.^[^ ^23^ ^]^ The macromolecules in the complex coacervates are held together by noncovalent interactions like electrostatic interactions, hydrophobic interactions, and hydrogen bonding.^[^ ^24^ ^]^ The physical properties and viscoelasticity of complex coacervates, that ultimately define their adhesive behavior, are closely linked to the strength and dynamics of the molecular interactions involved between the macromolecules. Dynamics refers to the time‐dependent behavior and movement of macromolecules within the complex coacervate. These properties can readily be influenced by a number of variables, detailed below and summarized in Figure 1B.

First of all, the intrinsic characteristics of the macromolecules that combine to form the complex coacervate play an important role in the resulting material properties. Intrinsic characteristics are defined as the inherent properties of the material itself, independent of external conditions. The polymer chemistry of the chains is an important factor impacting the properties of complex coacervates. This is because the functional groups on the polymer determine the type and strength of the interactions between the chains.^[^ ^24^ ^]^ For example, adding more hydrophobic groups to polyelectrolytes will make them more readily undergo coacervation, as there will be more hydrophobic interactions.^[^ ^25^ ^]^ The backbone of polymers also affects their interactions. For instance, the presence of a methyl group on the backbone of a polymer affects the phase behavior and rheology (the flow and deformation) of the resulting coacervate.^[^ ^26^ ^]^ Molecular weight also strongly affects the mechanical behavior of complex coacervates. Increased molecular weight promotes non‐covalent associations as well as chain entanglements, leading to materials that exhibit slower dynamics, or longer relaxation times, and consequently a more solid‐like behavior with a reduced ability to flow.^[^ ^27^ ^]^ In a similar way, an increase in the quantity of electric charge along the macromolecules (charge density) also leads to stronger electrostatic interactions, resulting in longer relaxation times and solid‐like properties.^[^ ^26^, ^28^ ^]^ In most cases, it was reported that a balanced ratio between positive and negative charges leads to higher interaction strength between the oppositely charged polyelectrolytes.^[^ ^29^, ^30^ ^]^ Furthermore, chain topology (the structural arrangement of polymer chains, e.g., linear, branched, looped) has been shown to influence the final viscoelastic complex coacervate properties.^[^ ^31^, ^32^ ^]^

Second, not only the macromolecules themselves, but also the environment in which the complex coacervates are prepared has a drastic impact on the phase behavior and material properties. These environmental parameters are defined as extrinsic factors. First of all, the presence of salt in the environment strongly impacts the properties of complex coacervates.^[^ ^33^, ^34^, ^35^ ^]^ Upon increasing salt concentration, polymer‐polymer electrostatic interactions are progressively replaced by polymer‐salt interactions, which drastically increases the mobility of the chains and their ability to take up water. Many complex coacervates can transition from rigid solids, to soft rubbery gels, to viscous liquids, due to this decrease in electrostatic interaction density.^[^ ^33^, ^36^ ^]^ If the salt concentration is increased further, the driving force for association between polyelectrolytes is too low, and no LLPS is observed.^[^ ^19^ ^]^ The point where no LLPS is observed anymore is known as the salt resistance of a complex coacervate.^[^ ^37^ ^]^ The effect of salt is strongly dependent on the type of polyelectrolytes in the coacervate, as well as the type of salt.^[^ ^38^, ^39^ ^]^ For example, divalent salts like CaCl_2_ have a much stronger plasticizing effect, making the material more fluid‐like, compared to monovalent salts like NaCl.^[^ ^34^ ^]^ Although more commonly associated with electrostatic interactions, ionic strength can also affect hydrogen bonding and hydrophobic interactions.^[^ ^40^ ^]^ An increased ionic strength leads to a decrease in hydrogen bonds, yet also to stronger hydrophobic interactions.^[^ ^40^ ^]^

The pH of the environment also exerts an effect on complex coacervates.^[^ ^41^ ^]^ Weak polyelectrolytes are macromolecules of which the charge present on the macromolecule is a function of the pH. As mentioned, the number of charges on the polyelectrolyte, as well as the ratio between positive and negative charges has a large effect on complex coacervates. For example, hyaluronic acid – chitosan complex coacervates behave as viscoelastic liquids at pH 4, while they form an elastic gel at pH 6.^[^ ^42^ ^]^ This effect is seen not only due to a change in electrostatic interactions, but also because pH influences the formation of hydrogen bonds.^[^ ^42^ ^]^

Temperature only has a limited effect on complex coacervates. Temperature influences the molecular interactions in coacervates in different manners; while increasing temperature strengthens hydrophobic interactions, it weakens hydrogen bonds.^[^ ^43^, ^44^ ^]^ Moreover, higher temperatures lead to faster relaxation of complex coacervates, favoring flow of the material.^[^ ^45^ ^]^ Additionally, it was found that the amount of complex coacervate that formed decreased when temperature was increased from 25 to 45 °C.^[^ ^46^ ^]^ More on the effect of temperature on the dynamics of complex coacervates has been described in a review article by Lalwani et al.^[^ ^12^ ^]^

Environmental conditions can also be altered *after* preparation of the complex coacervate, triggering a change in the material properties of the complex coacervate. This gives complex coacervates stimuli‐responsive properties (Figure 1C). As an example, complex coacervates can undergo a “salt switch”, which is a liquid‐to‐solid transition as a result of a decrease in ionic strength. Complex coacervates prepared at a high salt concentration will release salt ions upon contact with an environment with a lower salt concentration, leading to stronger electrostatic interaction between oppositely charged polyelectrolytes, and consequently solidification.^[^ ^47^ ^]^ Moreover, complex coacervates can also be responsive to changes in the pH of their environment. An interesting case of pH‐responsive complex coacervates can be found in sandcastle worms, that are able to glue sand grains and pieces of shell together in a marine environment.^[^ ^48^ ^]^ Inside the secretory duct of the worm (pH < 6), the complex coacervate is liquid, and once excreted into the ocean (pH > 8), the material transforms into a porous, solid foam.^[^ ^49^ ^]^ Altering the molecular design of the polyelectrolytes can induce thermoresponsive properties of complex coacervates. For example, by grafting thermoresponsive poly(N‐isopropylacrylamide) (PNIPAM) onto oppositely charged polymers, a temperature‐induced solidification of the complex coacervate was observed.^[^ ^50^ ^]^

While complex coacervates are able to form hydrogels, there are a few key differences between complex coacervates and traditional hydrogel materials.^[^ ^51^, ^52^ ^]^ Hydrogels are typically not formed through LLPS but by chemically or physically cross‐linking polymer chains. This gives them a 3D network structure full of pores, which can absorb large amounts of water. This structure makes hydrogels very suitable for application in the field of regenerative medicine. They form scaffolds for cells to infiltrate and regenerate tissue lost after injury or disease.^[^ ^53^ ^]^ Adhesive hydrogels are therefore regularly applied to treat skin wounds.^[^ ^54^ ^]^ Though both soft materials, complex coacervates are often of a more liquid‐like nature, while hydrogels have a more solid‐like, elastic structure. Hydrogels are not inherently stimuli‐responsive, like complex coacervates, although they can be engineered to respond to physical, chemical, or biological cues.^[^ ^55^ ^]^

**Figure 2:**
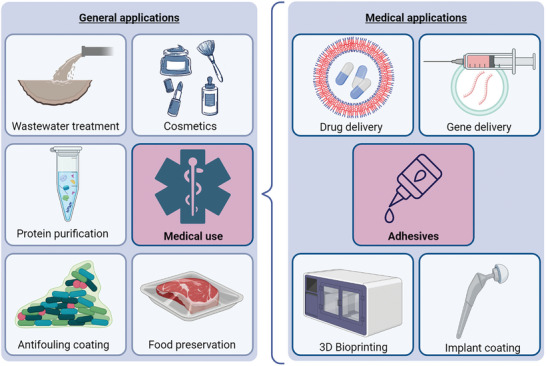
Summary of applications of complex coacervates, highlighting the current applications in medicine. Figure was created with BioRender.com.

The versatility of complex coacervates’ properties render them easily processable materials. Most commonly, they are prepared as viscous liquids, and their ability to transition into gels, solids, and foams upon environmental triggers is advantageously used for numerous applications (**Figure** **2**). Examples include wastewater treatment, protein purification, antifouling coatings, food preservation, and cosmetics.^[^ ^56^, ^57^, ^58^, ^59^, ^60^, ^61^ ^]^ Moreover, there are several emerging applications in the biomedical field.

An important use of complex coacervates in medicine is drug and gene delivery. By incorporating charged proteins, RNA, or DNA into a complex coacervate, their stability is improved for transportation and storage, and they are protected against degradation when administered as therapeutics.^[^ ^62^, ^63^ ^]^ Furthermore, micelles can be formed using complex coacervation, in which drugs can be encapsulated, enabling a controlled release and slower degradation of the therapeutic compounds.^[^ ^64^, ^65^ ^]^ Complex coacervate encapsulation is also a promising strategy for mRNA delivery, increasing the safety and stability for mRNA‐based therapies, such as vaccines against COVID‐19 and other viruses.^[^ ^66^ ^]^ Even CRISPR/Cas9 genome editing machinery can be encapsulated, ensuring higher transfection and editing efficiency.^[^ ^67^ ^]^ Additionally, complex coacervates have been shown to be a promising coating for medical implants, prohibiting bacterial growth while stimulating native cell proliferation on orthopedic and dental titanium implants.^[^ ^68^, ^69^ ^]^ Moreover, complex coacervates hold promise for 3D bioprinting of personalized implants.^[^ ^70^ ^]^ Lastly, complex coacervates are promising candidates for medical adhesives, which will be discussed in depth in the next section.

## 3 Complex Coacervate Medical Adhesives

**Figure 3:**
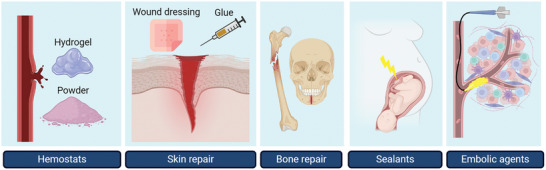
Different applications of complex coacervate medical adhesives. Figure was created with BioRender.com.

Biological tissues contain a large portion of water, ranging from 5–20% in adipose tissue to 80–83% in lung tissue.^[^ ^38^ ^]^ Therefore, a medical adhesive for application in such tissues should have strong adhesion properties under wet conditions. Complex coacervates exhibit low interfacial tension, enabling them to spontaneously spread on a submerged surface.^[^ ^16^ ^]^ They are weakly hydrophobic and immiscible with water, allowing them to remain at the application site without dissolving in fluids present like blood or mucus.^[^ ^23^ ^]^ In line with this, complex coacervates have been suggested to displace water from the substrate, maximizing their adhesive performance.^[^ ^71^ ^]^ Their liquid nature makes them easy to process and inject through syringes and catheters, avoiding the need for invasive surgical procedures for internal applications. Conventionally used cyanoacrylate adhesives are dependent on polymerization reactions that cause inflammation and damage.^[^ ^72^ ^]^ In contrast, complex coacervates can solidify upon encountering harmless environmental triggers like pH, temperature, and salt. Their adhesive and cohesive properties are mainly attributed to non‐covalent molecular interactions such as hydrophobic interaction, electrostatic interaction, and hydrogen bonding.^[^ ^10^ ^]^ Complex coacervates are suitable to be combined with additives, giving rise to the opportunity for drug‐loading.^[^ ^73^ ^]^ These properties make them suitable candidates for different classes of medical adhesives, detailed below and summarized in **Figure** **3**.

### 3.1 Hemostats

Uncontrolled internal bleeding resulting from surgery, trauma, or underlying medical conditions poses a significant risk to patient outcomes and survival.^[^ ^74^ ^]^ Acute hemostasis is needed to control bleeding, which is traditionally achieved using a tourniquet, gauze, or sponge. However, these techniques cannot be applied for internal bleeding as prolonged application of pressure is required.^[^ ^75^ ^]^ Hydrogels and hemostatic powders are an alternative that could be used for internal wounds, as they can be applied directly to the site of injury and do not need compression. A hemostatic adhesive should not dissolve in water or blood. It should exhibit strong wet adhesion, as it is required to remain at the application site, even under high pressures. Optimally, it should induce rapid hemostasis, for example by platelet activation and activating the coagulation pathway, which are important steps that lead to the formation of a blood clot. Currently available hydrogels, unfortunately, do not have sufficient adhesion to tissue in the presence of blood, and conventional powders cannot form a proper scab and may even dissolve in blood, failing to stop the bleeding.^[^ ^76^ ^]^ Since complex coacervates show a strong capability to displace interfacial fluid and bind to wet surfaces, they are excellent candidates when it comes to controlling internal bleeding. Several studies have investigated the hemostatic abilities of complex coacervates, both as hemostatic powders and as hydrogel biomaterials.

A complex coacervate powder, made out of the oppositely charged polymers polyethyleneimine (PEI) and polyacrylic acid (PAA), was able to form a bio‐adhesive hydrogel when brought into contact with wet tissues, making it a potential hemostatic material.^[^ ^77^ ^]^ Nevertheless, it was not able to absorb large amounts of water, a property required to stop active bleeding. To improve the properties of the PEI‐PAA adhesive hydrogel and make it better suited for hemostasis, quaternized chitosan (QCS) was added as a third component to the powder.^[^ ^76^ ^]^ Chitosan and its derivatives have been widely applied in the biomedical field due to their ability to absorb large amounts of water and to attract blood cells and platelets, enhancing coagulation.^[^ ^78^ ^]^ Chitosan can adhere to tissue through the formation of hydrogen bonds.^[^ ^79^ ^]^ The powder formed a strong adhesive layer within seconds upon coming into contact with bloody tissue, forming a strong physical barrier while attracting coagulation factors. Deposition onto bleeding wounds stopped massive hemorrhage in vivo in rat liver, heart, femoral artery, and tail, as well as in pig liver and spleen.^[^ ^76^ ^]^ Similarly, complex coacervate powders consisting of PAA and chitosan were able to significantly reduce blood loss in vivo in a severe liver and heart injury rat model.^[^ ^8^ ^]^ The main challenge with chitosan is its poor solubility and relatively weak mechanical properties under physiological conditions.^[^ ^80^ ^]^ Chitosan‐based coacervates could be further optimized by chemical modifications such as quaternization and phosphorylation, making them more suitable for biomedical applications.

Studies have also investigated complex coacervate powders containing tannic acid (TA), which is naturally derived and biocompatible, and can form cross‐links between polymers and biological surfaces.^[^ ^81^ ^]^ TA mixed with polyethylene glycol (PEG) and a large triblock co‐polymer, formed a powder which underwent complex coacervation through hydrogen bonding upon contact with water. At 37 °C, the complex coacervate then rapidly solidified, forming a biomimetic clot at the site of injury.^[^ ^82^ ^]^ TA mixed with gelatin and polyvinyl alcohol (PVA) led to the formation of a similar powder complex coacervate, also through hydrogen bonding.^[^ ^83^ ^]^ This immediately underwent gelation when it came into contact with a fluid, like a bleeding tissue, absorbing moisture and concentrating coagulation factors to induce clotting. Both TA‐based powders were able to induce rapid hemostasis, resulting in minimal blood loss in rat liver and tail injuries.^[^ ^82^, ^83^ ^]^

TA combined with biocompatible poly(N‐hydroxyethyl acrylamide), also undergoes complex coacervation through hydrogen bonding, forming an adhesive hydrogel.^[^ ^84^ ^]^ This hydrogel was also shown to have self‐healing and antibacterial properties, and good hemostatic ability, preventing blood loss in rat liver and tail injuries. Similarly, TA was able to form complex coacervate hydrogels with PAA, yielding good biocompatibility and hemostasis in a rat model.^[^ ^7^ ^]^ The strong, water‐resistant adhesion and cohesion of the material can be attributed to the multiple hydrogen bonds present.^[^ ^85^ ^]^ When a nano‐clay cross‐linker was introduced to this complex coacervate, adhesion was even further increased, going hand in hand with superior hemostatic performance.^[^ ^86^ ^]^ Another complex coacervate based on hydrogen bonding, composed of silico‐tungstic acid and PEG, showed similar results, including good adhesion and a significant reduction in bleeding in a rat liver model.^[^ ^87^ ^]^

A limitation of some of the synthetic materials used in these hemostatic materials is their biodegradability. Ideally, after the bleeding has stopped and the body has repaired the tissue at the site of injury, the hemostatic material should degrade. For example, the speed and total degradation of PAA is strongly dependent on its molecular weight, with high molecular weight PAA being very poorly degradable.^[^ ^88^ ^]^ Despite this fact, PAA‐based coacervate hemostats have not been optimized for their degradability, and long‐term effects have not been studied.^[^ ^8^, ^76^ ^]^ The same can be said for PVA‐based coacervate hemostats, as PVA is only degraded in the presence of specific microorganisms and degradation has not been detected in the body.^[^ ^89^, ^90^ ^]^

Protein‐based complex coacervates for hemostasis have also been explored, to avoid introducing foreign polymers in the recipient. Complex coacervate hydrogels made out of poly‐γ‐glutamic acid and oppositely charged lysozyme showed ultra‐high adhesion to wet and biological surfaces and strongly reduced blood loss after liver and tail injury in rats.^[^ ^91^ ^]^ It has been shown that extracellular matrix (ECM)‐derived proteins play an important role in physiological hemostasis.^[^ ^92^ ^]^ To this end, ECM proteins such as elastin have also been incorporated into hemostatic complex coacervates. Elastin‐based positively charged poly‐peptides formed a strong glue when complexed with negatively charged surfactants, which was able to stop liver and kidney bleeding in a rat model.^[^ ^93^ ^]^ Moreover, a collagen‐based complex coacervate hydrogel, also containing TA, was found to be an ultra‐stretchable adhesive, able to completely seal severely bleeding rat liver and hearts, even after a 7‐day follow‐up.^[^ ^94^ ^]^ Regular protein‐based adhesives carry the risk of allergic reactions and disease transmission, as the materials are naturally derived from, e.g., human plasma, or animal serum.^[^ ^95^ ^]^ These coacervate studies appear to overcome this limitation by using recombinant, bio‐mimetic proteins instead, creating strong and biocompatible adhesives.

To conclude, complex coacervate powders and hydrogels are excellent candidates for novel hemostatic agents. They are easy to apply, making them suitable for non‐compressible injuries. Their ability to strongly adhere to wet tissue has been shown to be beneficial for bleeding control. Both synthetic and natural polyelectrolytes can form complex coacervates that significantly inhibit blood loss in several types of organ injuries. While animal studies showed good hemostatic performance, it is important to optimize the degradability of the adhesives based on the application. Furthermore, clinical studies in humans have yet to be performed, which will be the next step toward advancing these materials from laboratory testing to clinical use.

### 3.2 Skin Repair

Wounds in the epidermis layer of the skin can recover within 1–2 weeks without any intervention, through the migration of new epithelial cells to the surface. However, when the underlying dermis layer is also damaged, repair may take up to 6 weeks, as the formation of granulation tissue is needed to fill the damaged area before re‐epithelialization can occur.^[^ ^96^ ^]^ This makes the body more vulnerable to infection at such sites. In order to aid the closure of extensive skin wounds, sutures, glue, or wound dressings can be used.^[^ ^97^ ^]^ Suturing is most commonly used, but sutures are invasive and can cause secondary damage or infection.^[^ ^97^ ^]^ Available cyanoacrylate glues are associated with toxicity, and fibrin‐ and albumin‐based glues are associated with weak strength and allergic reactions.^[^ ^2^, ^4^ ^]^ Hydrogel wound dressings developed in recent years are used because of their toughness and good retention of moisture. However, the required cross‐linking still leads to a degree of cytotoxicity, and their weak adhesion can lead to separation from the wound and may increase the susceptibility to infections.^[^ ^98^, ^99^, ^100^ ^]^ Ideally, an adhesive for skin repair should be flexible and elastic, maintaining adhesion during movement without causing discomfort to the patient. Apart from preventing infection, it should also minimize scarring. The low cytotoxicity and good adhesive properties of complex coacervates make them interesting to study in the context of wound healing, both as a glue and as a wound dressing.

Complex coacervates have demonstrated significant potential for facilitating wound healing. Notably, some complex coacervates that were studied for their hemostatic properties have also been investigated for their wound healing capacity.^[^ ^7^, ^76^, ^83^, ^91^, ^93^ ^]^ Complex coacervate glue made from elastin‐based peptides mixed with oppositely charged surfactant facilitated wound healing and skin regeneration in an in vivo 9‐day wound‐healing experiment in rats, surpassing the performance of sutures and a commercial medical adhesive.^[^ ^53^ ^]^ Additionally, this adhesive exerted an anti‐inflammatory effect by decreasing the levels of inflammatory cytokines interleukin 6 (IL‐6) and tumor necrosis factor alpha (TNF‐α) in the wound. Furthermore, two PAA‐based complex coacervate hemostats were used as wound dressings in circular injuries in rats. Here, they were shown to accelerate the wound closure rate, as well as induce the formation of new blood vessels (neovascularization) and reduce scarring.^[^ ^7^, ^76^ ^]^ Of interest are the complex coacervate hemostats that were also studied in the context of infected wound healing, as infection prolongs the wound healing process and can lead to severe complications like sepsis and organ failure.^[^ ^101^ ^]^ Complex coacervate hydrogels made of poly‐γ‐glutamic acid and lysozyme were able to stimulate repair of wounds within 15 days in a *S. aureus* infected rat model, outperforming a commercial wound dressing.^[^ ^91^ ^]^ Due to its excellent antimicrobial performance, the group treated with this complex coacervate showed the most neovascularization and even the return of hair follicles. Similarly, complex coacervate powder consisting of gelatin, TA, and PVA improved the healing process in MRSA‐infected wounds in a rat model.^[^ ^83^ ^]^ The powder could significantly reduce the number of bacteria present in the wound, accompanied by accelerated healing, compared to commonly used gelatin sponges and a commercial wound dressing. It is important that these materials are studied for their biodegradation, as little is mentioned about the amount of time they remain at the site of the wound. The speed of degradation that is required could depend on the size and type of wound, as they have different healing times.

A complex coacervate glue made of gelatin, TA, and PEG was also proposed as a novel adhesive for skin wound repair.^[^ ^102^ ^]^ The gel‐like glue was used to close incisions in a rat model, where it was able to significantly reduce the wound area in 7 days compared to sutures and a commercial tissue adhesive. This is in line with the increased collagen deposition and neovascularization in the group treated with complex coacervate. Furthermore, complex coacervate hydrogels composed of PEI, paired with either PAA or TA, were found to be suitable to be loaded with epidermal growth factor (EGF).^[^ ^51^ ^]^ EGF is known to be involved in re‐epithelialization and formation of granulation tissue.^[^ ^103^ ^]^ The EGF‐loaded hydrogels formed an adherent wound dressing, which accelerated wound healing and neovascularization in a rat model.^[^ ^51^ ^]^ Interestingly, the hydrogels made with TA were found to be more effective than the PAA‐gels, suggesting TA is more beneficial to use for wound healing purposes. This study highlights the opportunity for complex coacervate adhesives to be loaded with proteins, increasing their therapeutic potential. A limitation of the use of PEI in adhesive materials is its cytotoxic effect; it is known to exhibit some toxicity toward different cell lines.^[^ ^104^ ^]^ To further improve these complex coacervates, PEI cytotoxicity could be decreased by decreasing the molecular weight or increasing the degree of branching.^[^ ^105^ ^]^

Hyaluronic acid (HA) is a major component of the ECM and plays a large role in tissue regeneration, re‐epithelialization, and angiogenesis. This is why it has been thoroughly investigated in the context of wound healing.^[^ ^106^ ^]^ As it is a polyelectrolyte, it can be used to form complex coacervates. One example is when HA is combined with lysozyme, it forms a complex coacervate. This coacervate is able to reduce the wound size in a mouse model by 70% within 5 days, while a commercial dressing only reached 30% healing at this timepoint.^[^ ^100^ ^]^ The complex coacervate has self‐healing properties, meaning it is able to recover to its original state after deformation or fracture. Furthermore, it was also able to reduce the level of pro‐inflammatory TNF‐α while increasing vascular endothelial growth factor (VEGF).^[^ ^100^ ^]^ HA has also been combined with biopolymer chitosan to form a complex coacervate adhesive, showing one of the highest adhesion strength values for water‐rich complex coacervate underwater adhesives.^[^ ^107^ ^]^ The HA – chitosan coacervate was also studied in vivo, where it was found to accelerate wound healing in a rat model.^[^ ^108^ ^]^ This effect was even stronger when honey was added to the complex coacervate, triggering the stimulation of fibroblasts and the expression of VEGF. Although honey is known for its antibacterial properties, a challenge for its application is the lack of consistency of the material, depending on the location and species of bees, and production methods.^[^ ^109^ ^]^ To optimize this system, a synthetic type of honey could be developed. This may also help to make the treatment usable for patients with an allergy to bees or pollen. While HA‐based coacervates are promising for skin healing, their application may be limited to small wounds only. This is due to the rapid degradation in vivo through enzymatic activity and reactive oxygen species, which may result in insufficient support for the wound over the necessary healing period.^[^ ^110^ ^]^

In summary, complex coacervates have a high potential to be clinically used in the context of wound healing, both as glue and as wound dressings. By stimulating fibroblasts to deposit collagen, inducing neovascularization, and reducing inflammation, they often exceed the performance of commercial glues and dressings. Further optimization can be performed to improve degradation and cytocompatibility. No clinical trials have yet been performed or planned. These materials are interesting to investigate further, as they could improve wound care after injury and surgery.

### 3.3 Bone Repair

When a bone is damaged or fractured, osteoblasts can produce bone tissue to repair the structure.^[^ ^111^ ^]^ However, a large amount of bone loss due to traumatic injury or disease often requires orthopedic reconstructive surgeries, which involves attaching a bone substitute to the damaged area. Furthermore, bone damage to the face and skull often leads to the generation of large quantities of bone fragments that are difficult to repair using traditional methods like pins, plates, or screws.^[^ ^112^ ^]^ Therefore, using an adhesive can be a useful tool in bone repair. Ideally, the adhesive should support cell attachment, proliferation, and differentiation, promoting bone growth and healing. Moreover, significant bone loss is often accompanied by the presence of large amounts of blood, which means that the adhesive needs to have water‐resistant properties. As complex coacervates are immiscible with water, they are excellent candidates for this application.

Previously, the composition of the adhesive produced by the sandcastle worm was mimicked to produce novel bone adhesives.^[^ ^6^, ^113^, ^114^, ^115^ ^]^ Already in 2010, the first complex coacervate adhesives were tested in an in vivo cranial fracture rat model.^[^ ^115^ ^]^ The complex coacervate adhesive increased bone formation and prevented misaligned bone formation.

More recently, complex coacervates produced from recombinant mussel adhesive proteins (rMAP) and HA were combined with bone mineral particles as a novel approach to bone adhesion.^[^ ^9^ ^]^ The catechol groups in rMAP lead to strong tissue adhesion through binding to various nucleophiles in proteins.^[^ ^116^ ^]^ In a rat bone defect model, the group treated with the complex coacervate showed more newly formed bone tissue, compared to the group treated with bone mineral particles alone.^[^ ^9^ ^]^ The bone healing properties of complex coacervate alone, without the addition of bone mineral particles, were not tested in this study. Another composite material, consisting of a cellulose‐polyphosphodopamide – chitosan complex coacervate and mineral nanoparticles, was found to have stronger adhesion to bone tissue when compared to commercial bone adhesives.^[^ ^117^ ^]^ The material contributed to successful sternum bone healing in an in vivo rabbit model due to significant new bone formation and collagen deposition, while minimizing the formation of fibrotic tissue.

Modified PEI and chondroitin sulfate, of which the latter is derived from cartilage, are also able to undergo complex coacervation to form an adhesive.^[^ ^118^ ^]^ This material showed high lap shear strength on cartilage and was also tested in a cartilage defect rat model, demonstrating its biocompatibility and feasibility for use for cartilage repair. However, the in vivo results are not very extensive and require follow‐up before advancing to clinical trials.

Interestingly, limited data is available on the use of complex coacervates as bone adhesives when compared to other applications, e.g., skin applications. Potentially, complex coacervate adhesives are more suitable for soft and elastic tissues, as they typically exhibit these same soft and elastic material properties, whereas bone and cartilage exhibit a much higher stiffness. The few studies that have investigated the application of complex coacervates in bone and cartilage showed promising results in vivo. However, the maximum follow‐up period for these studies is only 4–12 weeks, while bone remodeling is an extensive process that can take months to years.^[^ ^119^ ^]^ Therefore, especially for bone healing applications, it is important to perform long‐term studies on these complex coacervate adhesives.

### 3.4 Sealants

Sealants are used to form a physical barrier to prevent leakage of fluids or air. They can be used to treat defects in organs or to cover suture holes in tissues.^[^ ^16^ ^]^ Sealants should be impermeable and resistant to pressure and provide flexibility to accommodate for movement. Currently available naturally derived sealants are used in a wide array of applications, including repair of hernias, colon defects, and in thoracic surgeries.^[^ ^16^ ^]^ Additionally, synthetic sealants, such as PEG and cyanoacrylates, are being studied for applications such as preventing air leaks after pulmonary surgery, and preventing leakage of cerebrospinal fluid after cranial surgery. Similarly to natural sealants, they do not provide enough strength to be used on their own, without the additional use of sutures.^[^ ^16^ ^]^

While research into using complex coacervates as medical sealants is limited, there are several interesting studies describing their application as sealants. Using complex coacervate sealants to treat defects occurring during pregnancy is one of these applications. Early during pregnancy, an incomplete closure of the spine can occur (spina bifida). Spina bifida can lead to physical problems like leg paralysis and bladder control problems, as well as neurological problems like Attention Deficit Hyperactivity Disorder (ADHD) and learning difficulties.^[^ ^120^ ^]^ *In‐utero* repair can reduce morbidity and mortality compared to postnatal repair, but often still comes with long‐term complications.^[^ ^121^ ^]^ In a sheep model, spina bifida was treated using biocellulose patches with a complex coacervate consisting of synthetic poly(acrylamide‐co‐aminopropyl methacrylamide) and poly (2‐(methacryloyloxy)ethyl phosphate dopamine methacrylamide.^[^ ^121^ ^]^ However, this adhesive did not stay at the defect site, leaving the spina bifida unrepaired. The same adhesive has also been studied for the repair of defects in fetal membranes.^[^ ^122^ ^]^ In an in vitro human uterine model, a defect was generated and sealed using a fetal membrane patch and the above‐mentioned complex coacervate. The complex coacervate displayed good adhesion and prevented fluid leakage in this model without showing any signs of cytotoxicity. Therefore, this adhesive was further studied in an in vivo pregnant swine model.^[^ ^123^ ^]^ However, this turned out to not be an appropriate model to study fetal membrane repair, as swine fetal membranes can heal spontaneously, and therefore no differences were observed between treated and untreated groups. Furthermore, there was an apparent increase in fetal loss in the animals treated with the complex coacervate. Further applications in pregnancy defects have not been studied since, and these mostly negative results do not show much promise of utilizing complex coacervates as sealants in this field of research.

More promising results have been found studying the application of complex coacervates for urinary fistula sealing. A urinary fistula is an abnormal opening in the urinary tract, leading to constant leakage of urine or feces.^[^ ^124^ ^]^ Current treatments are surgical, which are invasive, painful, and do not completely stop fluid leakage.^[^ ^125^ ^]^ Therefore, an adhesive sealant could be a promising alternative, but no available product has been found to be suitable for this application yet. A complex coacervate composed of rMAP and HA was tested and had strong underwater adhesion to bladder tissue.^[^ ^126^ ^]^ Moreover, the pressure needed for the adhesive to fail and induce leakage was measured in an ex vivo rat bladder, in which the complex coacervate sealant outperformed commercially available adhesives after 12 h of incubation. Further studies are required to confirm these results in vivo. An important limitation is the biodegradability of this material. Once the material will degrade, the sealant will likely start to leak and lose its function. Therefore, biopolymers like rMAP and HA may not be the most optimal materials for this application. It could be of interest to study complex coacervates consisting of synthetic, non‐degradable polymers in the context of urinary fistula sealing.

Lastly, complex coacervate sealants have been investigated in the context of gastrointestinal perforations. A hole in the esophagus, stomach, or intestines can be caused by traumatic injury or disease and can eventually lead to life‐threatening sepsis.^[^ ^127^ ^]^ To repair the defect and remove necrotic tissue, surgical intervention is nearly always necessary. A complex coacervate powder, consisting of PEI and PAA, was developed to repair gastrointestinal perforations in a less invasive manner.^[^ ^77^ ^]^ The powder rapidly formed a hydrogel upon contact with water, forming a strong adhesive on porcine intestine and stomach tissue. The strong adhesion was reported to be an effect of absorption of interfacial water and diffusion of the polymers into the substrate tissue. When used to seal an ex vivo porcine intestine and stomach, the powder showed higher bursting pressure compared to a commercial sealant. Furthermore, the sealant was able to decrease gastric perforation size in an in vivo rat model by promoting re‐epithelialization and neovascularization, while decreasing inflammation. Therefore, the PEI/PAA powder is able to seal and promote healing of gastric perforations in vivo.^[^ ^77^ ^]^ When studying gastric applications, the extremely low pH of the environment should be taken into account. The charge density of the polymers used in this study is pH dependent, and it was found that the formed hydrogel could swell and dissolve at such extreme pHs.^[^ ^77^ ^]^ Perhaps, using strong polyelectrolytes, of which the charge density is independent of pH, could overcome this problem and improve the material.

While spina bifida and fetal membrane repair do not seem appropriate applications for complex coacervate sealants, urinary fistulas and gastrointestinal perforations are interesting potential applications. However, more in vivo studies, possibly in larger animals, are required for future clinical translation. Furthermore, many other applications have yet to be explored, such as for air leakage in the lungs, or the repair of hernias and pancreatic fistulas.

### 3.5 Embolic Agents

Embolization is a technique by which blood flow is blocked in a controlled and minimally invasive manner. This intervention is used to treat tumors, control hemorrhage, or exclude aneurysms.^[^ ^128^ ^]^ For occluding large blood vessels, metal coils or gelatin foam are commonly used, while smaller vessels are treated with liquid embolic agents, like alcohols and detergents.^[^ ^129^, ^130^ ^]^ One common challenge for the development of liquid embolic agents is viscosity, as the formulation needs to be of low viscosity in order to be delivered through narrow catheters. Furthermore, the embolic agent should be immiscible with blood to ensure it remains at its application site. The material should be strong enough to withstand the hemodynamic forces within the vessel without dislodging or fragmenting. Although rapid development has taken place over the past years, currently available liquid embolic agents are still associated with risks of vasospasm, tissue necrosis, and irreversibly gluing the delivery catheter to the embolization site.^[^ ^131^ ^]^

A complex coacervate liquid embolic agent was first described in 2016, where polyphosphate (PP) formed complex coacervates with divalent cations like calcium, strontium and barium.^[^ ^39^ ^]^ PP is present in human platelets and is important in coagulation, which makes it an interesting candidate for an embolic agent.^[^ ^132^ ^]^ The liquid embolic agent consists of two components, PP solution and cation solution, which are mixed inside the needle at delivery. Upon injection into the auricular artery in an in vivo rabbit model, the embolic agent formed a solid complex coacervate, successfully occluding the artery.^[^ ^39^ ^]^ In a follow‐up study, it was found that this formulation could be loaded with doxorubicin, a chemotherapy drug used to treat several types of cancer.^[^ ^133^ ^]^ Although its beneficial effects have been demonstrated in vitro, it has yet to be tested in vivo. As soluble barium is toxic, it is crucial to study barium release from the material during long‐term degradation. Another complex coacervate, consisting of QCS and gum Arabic (GA), has been loaded with doxorubicin.^[^ ^134^ ^]^ In a rat model, this complex coacervate was able to successfully embolize the renal arteries, as well as the arteries supplying tumors in the ears and liver. The tumors embolized with the doxorubicin‐loaded QCS/GA complex coacervate significantly reduced in size compared to the empty complex coacervate, showing its effectiveness against tumors in rabbit ears and livers.^[^ ^134^ ^]^ A longer follow‐up is required to determine the true anti‐tumor efficacy, as the animals were only observed for 7 days after embolization.

Moreover, a natural complex coacervate composed of salmine sulfate and phytic acid has been studied as a liquid embolic agent.^[^ ^131^ ^]^ The complex coacervate was found to be injectable through long and narrow catheters used for embolization. In an in vivo rabbit model, it was able to fully embolize renal arteries without any migration of the material. There were no adverse reactions observed in the targeted tissue. Building on this research, an embolic agent prototype named GPX was developed, consisting of synthetic polyguanidium and inorganic PP. First, it was tested to see whether GPX could be used as a treatment for aneurysms in rabbits.^[^ ^135^ ^]^ Here, it was shown that the material was able to fully occlude aneurysms, and this effect was still seen 1 month after treatment. However, a foreign body reaction to GPX was also detected after 1 month. Nevertheless, GPX was further investigated in a swine model, in which it also successfully occluded the renal artery and sub‐branches without adhering to the delivery catheter.^[^ ^136^ ^]^ Lastly, it has been shown that GPX can be loaded with contrasting agents or doxorubicin.^[^ ^136^ ^]^

Very recently, the GPX Embolic Device (Fluidx, Salt Lake City, Utah) was used in a First‐in‐Human trial, making it the only complex coacervate adhesive that has thus far made it to clinical trials.^[^ ^137^ ^]^ The study had a 30‐day follow‐up, and 17 patients with various pathologies were enrolled. In all cases, the target regions were successfully occluded. Two patients displayed severe adverse events potentially related to the device directly after the procedure. However, these events were resolved at the 7‐day follow‐up. Other mild and moderate adverse events that were reported were headache, sinus infection, atrial fibrillation, and hematuria.^[^ ^137^ ^]^ This first clinical trial showed good technical success, short‐term durability, and relatively low adverse events, showing promise to be used clinically in the future. Larger studies with longer follow‐up will be required to further investigate the safety and effectiveness of this material. The foreign body reactions noted in earlier animal studies warrant close monitoring in future trials.

## 4 Future Perspectives

Complex coacervates are currently investigated for various applications in the medical field, including their application as a medical adhesive. Through testing in various in vivo models, like rat, rabbit, and swine models, these adhesives have been shown to be promising candidates for clinical use. Complex coacervate adhesives can be used as hemostatic and embolic agents, adhesives for skin and bone repair, and as sealants (Figure 3). However, only one clinical trial in humans has been performed to date, in which the complex coacervate GPX Embolic Device was shown to be feasible, effective, and safe as an embolic agent. It is expected that in the coming years, several other complex coacervates will be tested in clinical trials for a plethora of medical applications.

However, before complex coacervates are used in clinics, it is important to perform long‐term in vivo animal studies, as most studies to date investigating the effects of complex coacervates have used short‐term models with a maximum duration of 4–6 weeks. Especially in larger animals, a study period of one or two years would provide a better understanding of how complex coacervates interact with tissues. Such testing would provide insight into their degradation and migration over time, as well as their long‐term health effects. After this, more materials could move on to in‐human trials to confirm their beneficial effects. As mentioned, complex coacervate properties vary with varying temperature, pH, and osmolarity.^[^ ^47^, ^49^, ^50^ ^]^ Therefore, it is important to determine what will happen to the complex coacervate material if a patient has a fever or a local change in salt concentration or pH.

Furthermore, before moving to clinical application, upscaling the production of complex coacervates presents a large challenge that needs to be addressed. First of all, this depends on the type of macromolecules that form the complex coacervate. If complicated synthesis, modification and purification processes are required to produce the macromolecules, it will be more challenging to produce the adhesives at a large scale. High costs associated with production of specialized biopolymers or recombinant proteins may limit the commercial viability of these materials. Another large challenge in scaling up is achieving reproducible final products. Small changes in environmental production settings can already have large effects on the final properties of a complex coacervate, so a consistent environment is crucial. Moreover, additional research is required optimize sterilization methods for complex coacervates, to ensure patient safety.

Looking forward, there are many still unexplored applications of complex coacervate adhesives. For example, to stop the leakage of air in patients with pneumothorax by sealing the defect in the lung. Possibly, complex coacervates could play a beneficial role in nerve injuries, providing structural support during regeneration. Other possible additional applications which have not been described include hernia repair, sealing of several types of fistulas (e.g., pancreatic and enterocolic fistulas), and dental adhesion. In the field of bioelectronics, complex coacervates could be explored as adhesives to attach electronic components to tissues, such as biosensors or wearable health devices. Moreover, it would be interesting to further explore the combination of the use of complex coacervates as adhesives with their ability to be loaded with drugs, growth factors, or even stem cells, providing a more effective regenerative treatment.

To summarize, complex coacervates are widely studied as medical adhesives in the context of hemostatic and embolic agents, adhesives for skin and bone repair, and sealants. The biocompatibility and ability to adhere to wet tissues of many complex coacervates make them promising alternatives to currently available cytotoxic, synthetic adhesives and weak, natural adhesives. Longer‐term in vivo studies are required to advance the field to clinical trials and eventually use complex coacervate adhesives for large groups of patients.

## Conflict of Interest

The authors declare no conflict of interest.
